# The Impact of Climate Change on Metal Transport in a Lowland Catchment

**DOI:** 10.1007/s11270-017-3261-4

**Published:** 2017-02-17

**Authors:** René R. Wijngaard, Marcel van der Perk, Bas van der Grift, Ton C. M. de Nijs, Marc F. P. Bierkens

**Affiliations:** 1 Department of Physical Geography, Faculty of Geosciences, Utrecht University, P.O. Box 80115, 3508 TC Utrecht, The Netherlands; 2 FutureWater, Costerweg 1V, 6702 AA Wageningen, The Netherlands; 3 Department of Subsurface and Groundwater Systems, Deltares, Princetonlaan 6, 3584 CB Utrecht, The Netherlands; 4 National Institute for Public Health and the Environment (RIVM), P.O. Box 1, 3720 BA Bilthoven, The Netherlands

**Keywords:** Climate change, Metal transport, Lowland catchment, Modeling, Quickflow, Baseflow

## Abstract

This study investigates the impact of future climate change on heavy metal (i.e., Cd and Zn) transport from soils to surface waters in a contaminated lowland catchment. The WALRUS hydrological model is employed in a semi-distributed manner to simulate current and future hydrological fluxes in the Dommel catchment in the Netherlands. The model is forced with climate change projections and the simulated fluxes are used as input to a metal transport model that simulates heavy metal concentrations and loads in quickflow and baseflow pathways. Metal transport is simulated under baseline climate (“2000–2010”) and future climate (“2090–2099”) conditions including scenarios for no climate change and climate change. The outcomes show an increase in Cd and Zn loads and the mean flux-weighted Cd and Zn concentrations in the discharged runoff, which is attributed to breakthrough of heavy metals from the soil system. Due to climate change, runoff enhances and leaching is accelerated, resulting in enhanced Cd and Zn loads. Mean flux-weighted concentrations in the discharged runoff increase during early summer and decrease during late summer and early autumn under the most extreme scenario of climate change. The results of this study provide improved understanding on the processes responsible for future changes in heavy metal contamination in lowland catchments.

## Introduction

Since the Industrial Revolution, soil and water contamination by heavy metals [e.g., cadmium (Cd), copper (Cu), lead (Pb), and zinc (Zn)] has become an increasingly serious threat in many regions of the world (Nriagu 1996; Su et al. 2014). Due to human activities such as mining, ore smelting, fuel combustion, waste disposal, and agricultural practices, terrestrial and aquatic environments have been enriched by heavy metals (Nriagu and Pacyna 1988; Kjøller et al. 2004; Jeričević et al. 2012), which may have adverse impacts on ecosystem functioning and human health.

Biogeochemical processes, including sorption/desorption, complexation, dissolution/precipitation, and uptake/release by biota, control the mobility of heavy metals and thus the residence time in soils and water (Carillo-Gonzáles et al. 2006; Reeder et al. 2006). When introduced into the environment, heavy metals tend to accumulate in soils and sediments (Foster and Charlesworth 1996) because of their affinity for sorption processes. Therefore, it may take decades to centuries or longer before heavy metals leach to groundwater (Seuntjens 2002; Degryse and Smolders 2006; Bonten et al. 2012; Joris et al. 2014). The solid-solution partitioning in soils is controlled by pH, redox potential, clay and soil organic matter (SOM) content, and the concentration of complex organic or inorganic ligands and competing cations (Elliott 1986; Hornburg and Brümmer 1993; Sauvé et al. 2000; Römkens et al. 2004; Pédrot et al. 2008; Unamuno et al. 2009; Degryse et al. 2009; Acosta et al. 2011; Groenenberg et al. 2012). Dissolved heavy metals are transported to surface waters via several hydrological pathways. Metals are transported downward through the unsaturated zone to groundwater. In groundwater, the transport rate of metals depends on the groundwater recharge rate, drainage density, hydraulic conductivity, porosity, and physiochemical conditions (McNab et al. 2006). Heavy metals may also take shortcut pathways to surface waters via quickflow routes including overland flow and tube drain flow. This happens in particular when the precipitation intensity exceeds the infiltration capacity of soils or when soils are saturated (Smith and Goodrich 2006). Due to large accumulation of metals present in upper soil layers, metal concentrations are generally higher in water discharging to surface waters under quickflow conditions (Rozemeijer and Broers 2007; Bonten et al. 2012).

Future climate change is hypothesized to affect the hydrology of catchments (IPCC 2013). It is likely that evapotranspiration will increase due to atmospheric warming, and in addition, there is medium confidence that heavy precipitation events will become more frequent and intense over many areas of the globe towards the end of the twenty-first century (Seneviratne et al. 2012). These changes may affect the relative proportions of quick- and baseflow towards surface waters and, hence, the transport rates and pathways of heavy metals (Miller et al. 2003; Middelkoop 2008). For example, intensification of precipitation may result in larger proportions of quickflow, which could accelerate the leaching of heavy metals to surface waters.

The number of studies dedicated to the impact of climate change on water quality in catchments is limited in comparison with studies focusing on the relation between climate change and quantitative aspects of catchment hydrology. There have been few studies dedicated to the impact of climate change on various water quality aspects (e.g., Murdoch et al. 2000; Van Bokhoven 2006; van Vliet and Zwolsman 2008; Whitehead et al. 2008, 2009). For example, Van Bokhoven (2006) projected the water quality of the Rhine River to deteriorate as a consequence of the increased occurrence of hydrological extremes (i.e., droughts and floods). During floods, increases in heavy metal concentrations due to desorption or re-suspension are projected, while during droughts increases in eutrophication and heavy metal concentrations due to a decrease in dilution may be expected.

Studies on the effects of climate change on heavy metal transport are even sparser. Visser et al. (2012) assessed the effects of future climate change on the hydrology and leaching of Cd and Zn in the Keersop catchment, a lowland catchment located in the south of the Netherlands. At the end of the twenty-first century, lower concentrations of Cd and Zn were projected as a result of lower discharge and lower water levels caused by higher evapotranspiration rates and an associated slowing down of groundwater flow. On the other hand, Joris et al. (2014) showed that climate change results in an increase in the cumulative Cd leaching flux to groundwater in the nearby Kempen area in the Dutch-Belgian border region compared to a no-climate-change reference scenario. These seemingly contrasting results demonstrate that the effects of climate change on the transport of heavy metals in catchments is multifaceted, complex, and equivocal, and requires a thorough understanding of the hydrological dynamics and pathways.

The aim of this study is to quantify the impacts of future climate change on the transport of heavy metals (i.e., Cd and Zn) from soils to surface waters in contaminated lowland catchments. For this, we selected the Dommel catchment, in the border region of the Netherlands and Belgium, which has been contaminated by metal inputs from zinc smelters, as a case study area. A conceptual semi-distributed hydrological model was forced with future projections derived from the KNMI’14 climate change scenarios (KNMI 2015a). Subsequently, outputs from the hydrological model were used as input for a metal transport model, which simulates Cd and Zn concentrations and loads in the different hydrological pathways that are connected with surface waters.

## Methods

### Study Area

**Fig. 1:**
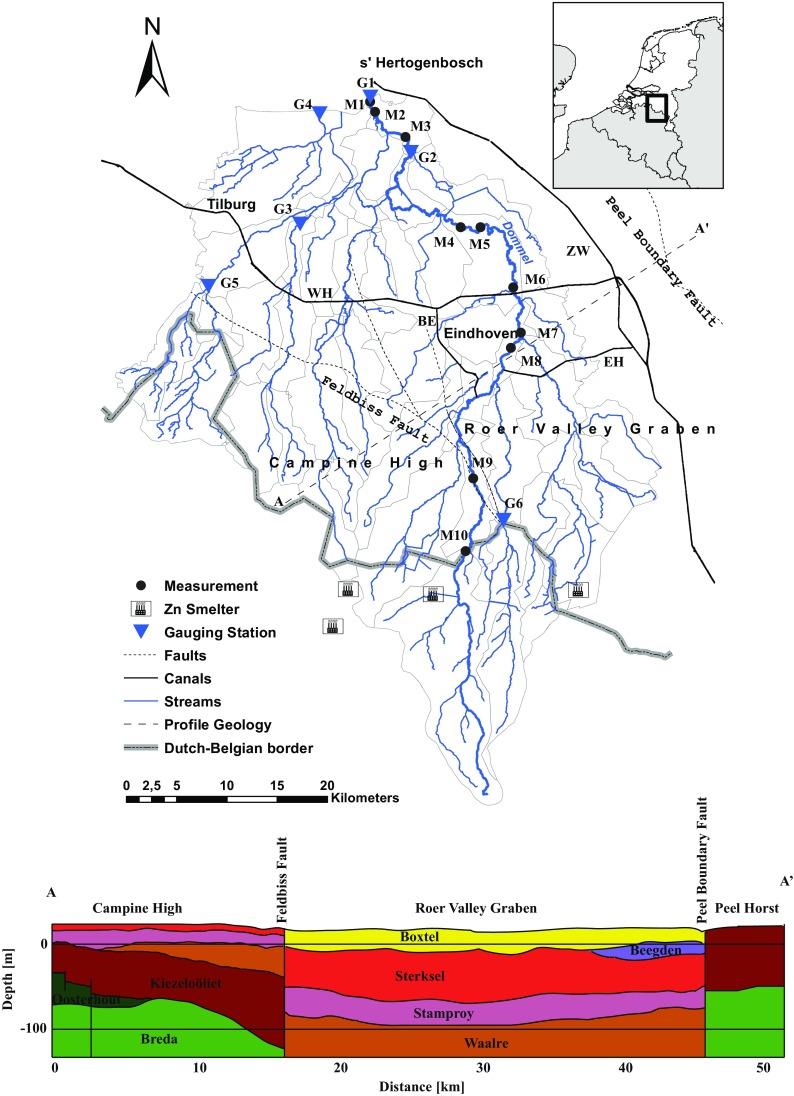
Study area Dommel catchment and the geological profile along the *dotted line* (*A*–*A*′). *M1*–*M10* represents the measurement locations where Cd and Zn locations have been measured. *G1*–*G6* represents the discharge gauging stations. *ZW* Zuid-Willemsvaart canal, *WH* Wilhelmina canal, *BE* Beatrix canal, *EH* Eindhoven canal. Sources: REGIS VII.1 (Vernes et al. 2005); VHA (AGIV 2014a); TOP10NL (Kadaster 2015)

This study focuses on the catchment of the Dommel River (Fig. 1). The main reason for choosing the Dommel catchment as study area is the severely contaminated state of the Kempen area, which encompasses roughly the southern half of the catchment in Belgium and the Netherlands. In the period 1880–1974, four zinc-ore smelters (three in Belgium, one in the Netherlands) emitted large amounts of heavy metals, such as Cd and Zn. Atmospheric deposition resulted in high and widespread accumulation of these metals in the upper soil layers surrounding the smelters (Van der Grift and Griffioen 2008; Bonten et al. 2012). In addition, ore slags from these smelters were used to pave roads and gardens, resulting in leaching of Cd and Zn from these roads to groundwater (Copius Peereboom-Stegeman and Peereboom 1989; Bonten et al. 2012). After 1974, atmospheric emissions have decreased drastically due to modifications in the production schemes of the ore smelters. Nevertheless, current Cd and Zn concentrations in upper soils are still high, threatening the quality of the terrestrial and aquatic environment (Van der Grift and Griffioen 2008; Visser et al. 2012).

The entire study area encompasses an area of about 1900 km^2^, of which the largest part is located in the Netherlands (∼1500 km^2^). The Dommel River has a total length of 146 km and runs from its source in the Kempen region to its confluence with the Meuse close to s’-Hertogenbosch, located downstream of Eindhoven (De Jonge et al. 2008) (see Fig. 1). The Dommel catchment consists of a natural system of brooks and streams, extended with a network of ditches and tile drainage that have been installed for agricultural purposes in the nineteenth and twentieth century (Visser et al. 2012). In addition, water levels in the Dommel catchment are controlled by the artificial supply of water from the Meuse River via the Zuid-Willemsvaart, Wilhelmina, Beatrix, and Eindhoven canals during dry periods in order to prevent agricultural damages (Vroege and Hoijtink 2013). In the Dommel catchment, elevation ranges from 80 m a.s.l. (above sea level) at the Kempen Plateau to 2 m a.s.l. close to the Dommel/Meuse confluence. The region experiences a humid and temperate climate with a mean temperature of 10.3 °C and a mean annual precipitation of 750 mm (Eindhoven meteorological station) (KNMI 2011). The prevailing soil types in the Dommel catchment are podzols (De Bakker and Schelling 1989). Geologically, the Dommel catchment can be divided into two regions: the *Campine High* in the southwest and the *Roer Valley Graben* in the northeast. The Roer Valley Graben is a region of active tectonic subsidence, bounded by the Feldbiss Fault (southwest) and the Peel Boundary Fault (northeast) (Schokker 2003; Petelet-Giraud et al. 2009). The upper geological unit in the Roer Valley Graben is the Late Pleistocene/Holocene Boxtel Formation characterized by sandy deposits alternated with thin loam and peat layers. The Boxtel Formation and the Middle-Pleistocene Beegden Formation belong to the upper aquifer in the Roer Valley Graben (i.e., with a thickness of approximately 100 m). The upper geological unit at the Campine High is the Middle-Pleistocene Sterksel Formation, which is characterized by coarse-grained fluvial deposits from the Rhine River. This formation belongs to the upper aquifer at the Campine High (i.e., with a thickness of 10–20 m). The underlying formations are (with increasing age) Early-Pleistocene Stamproy Formation, Early-Pleistocene Waalre Formation, Early-Pleistocene Maassluis Formation, Pliocene Kiezeloöliet Formation, Pliocene Oosterhout Formation, and the Miocene Breda Formation (De Mulder et al. 2003; Schokker 2003).

### Model Description

The effects of climate change on Cd and Zn transport in the Dommel catchment were investigated by using a semi-distributed rainfall-runoff model coupled to a metal transport model. The model system was set up in the PCRaster–Python environment (Karssenberg et al. 2010) with a spatial schematization of 250 × 250 m^2^. The hydrological and metal transport models reported at a daily resolution with a variable computational time step of minimal 1 h in order to assure numerical instability.

#### Hydrology

The WALRUS (Wageningen Lowland Runoff Simulator) model (Brauer et al. 2014a) was used to simulate current and future daily discharge in the Dommel catchment. WALRUS is a conceptual lumped hydrological model that has specially been developed for application in small lowland catchments. It accounts for the coupling between unsaturated and saturated zone, the feedbacks between ground- and surface water, and the wetness dependency of flow routes (Brauer et al. 2014a, b). The model consists of a quickflow (i.e., overland, macropore, and drainpipe flow), surface water, and a soil reservoir (including the saturated and unsaturated zone), and has five model parameters that require calibration. These parameters are the wetness parameter (*c*_*W*_), the quickflow reservoir constant (*c*_*Q*_), the vadose zone relaxation time (*c*_*V*_), the groundwater reservoir constant (*c*_*G*_), and the bankfull discharge (*c*_*S*_). For a more detailed description of WALRUS, we refer to the publications of Brauer et al. (2014a, b).

For large-sized catchments, such as the Dommel catchment, the application of WALRUS as a single model entity is not cumbersome due to the heterogeneous catchment characteristics (e.g., soil and drainage density) and the inability to account for the delay and attenuation of flood waves in the channels. For this reason, WALRUS was implemented and applied in a semi-distributed way, which was achieved in two steps:

1. The application of WALRUS for 44 delineated subcatchments.
2. The implementation of a routing module that routes the generated runoff using a kinematic wave approach (Chow et al. 1988).

#### Cd and Zn transport

A metal transport model was set up to simulate the transfer of Cd and Zn to the river network for the Dutch part of the Dommel catchment solely. In the Belgian part, the leaching and transfer of Cd and Zn was not simulated due to low data availability. The modeling approach comprised three steps: (1) the estimation of the loading towards the river network via quickflow, (2) the estimation of the loading towards the river network via baseflow, and (3) the estimation of mean flux-weighted concentrations in the runoff water discharged in the river network, based on the loading estimated in the former two steps.

For the estimation of the metal loading to the river network via quickflow and baseflow, the historic development of the topsoil Cd and Zn concentrations was first estimated. For this, we used maps Cd and Zn concentration in the topsoil for the year 1995 (Van der Perk et al. The response of metal leaching from soils on climate change and land management in a temperate lowland catchment, submitted), which were previously created by combining and interpolating Cd and Zn concentration measurements from various databases. The accumulation of Cd and Zn in the topsoil between the start of the operation of the zinc-ore smelters in 1880 and 1974 was interpolated using the atmospheric and agricultural Cd and Zn loads to soil as reported by Bonten et al. (2012). For the 1975–1995 period, the decline in Cd and Zn contents in the topsoil was modeled according to the metal leaching rates as simulated by Van der Perk et al. (submitted). This yielded annual values for the total Cd and Zn concentrations in the topsoil.

Second, a leaching model was applied to estimate the annual Cd and Zn concentrations in the soil leachate to groundwater for the period 1880–2100. For this, the upper 200 cm of the soil was subdivided into ten 20-cm-thick layers. The topsoil reactive concentrations were estimated from the total metal concentrations using the empirical partition-relations of Römkens et al. (2004):

$$ \log {Q}_{Zn}=0.428+0.183\ast \log S O M-0.298\ast \log clay+1.235 \log T{M}_{Zn} $$

$$ \log {Q}_{Cd}=0.289+0.022\ast \log S O M-0.062\ast \log clay+1.075 \log T{M}_{Cd} $$

where *Q* is the reactive metal content corresponding to the concentration of metals extracted with 0.43 M HNO_3_ (mol kg^−1^), *SOM* is the total soil organic matter content (%), clay is the clay content (%), and *TM* is the total metal concentration (mol kg^−1^). Subsequently, the metal concentrations in solution were estimated using the empirical partition-relations derived by Groenenberg et al. (2012):

$$ \log {C}_{Zn} = 0.93+0.99 \log {Q}_{Zn}-0.43 \log S O M-0.22 \log clay-0.14 \log A l F{e}_{ox}+0.12 \log DOC-0.46 p H $$

$$ \log {C}_{C d} = 1.60+1.11 \log {Q}_{C d}-0.62 \log S O M-0.39 \log A l F{e}_{ox}+0.29 \log DOC-0.41 p H $$

where *C* is the metal concentration in solution (mol L^−1^), *AlFe*_*ox*_ is the sum of oxalate extractable Al and Fe (mmol kg^−1^), and *DOC* is the dissolved organic matter concentration (mg L^−1^).

The SOM contents and DOC concentrations in the topsoil layer under present conditions and the climate scenarios were derived from the spatial implementation of the soil organic matter Century model (Metherell et al. 1993; Stergiadi et al. 2016; Van der Perk et al. submitted). The SOM contents and the other soil physicochemical parameters for the deeper soil layers, except DOC, were obtained from the STONE database (Van Bakel et al. 2008). DOC leaching through the successive soil layers was simulated using the soil water flux as modeled by the Century model. The biodegradation of the rapidly and slowly degradable DOC pools during downward transport was simulated using the double-exponential model reported by Kalbitz et al. (2003). The distribution of DOC across the two pools and their associated degradation rates for the various land use classes were also obtained from Kalbitz et al. (2003). The water residence time in the soil layers was calculated using the soil water fluxes and water content at field capacity, which was estimated based on the particle size distribution, SOM content, and soil bulk density using the empirical relations reported by Gupta and Larson (1979).

Like DOC, the leaching of Cd and Zn to groundwater was simulated using the water fluxes from the Century model. In each soil layer, the solid-solution partitioning was updated using the above empirical relations between the dissolved metal concentrations and the reactive metal concentrations. The metal concentrations in the leachate were calculated from the mean concentrations for the depth interval between average lowest and highest groundwater levels.

The annual Cd and Zn concentrations in the leachate were used to estimate the metal loadings towards the river network. The metal concentrations in quickflow were assumed to be equal with the concentrations in the leachate. For the estimation of metal concentrations in baseflow, the groundwater transit time distributions were taken into account. Under the assumption that the aquifer is isotropic and the Dupuit–Forchheimer assumption, the groundwater transit time can be estimated by (Mourad 2008)

$$ T= D\frac{n}{N}\left(\frac{x_2}{\varDelta x} \ln \left(\frac{X}{x_2}\right)-\frac{x_1}{\varDelta x} \ln \left(\frac{X}{x_1}\right)+1\right) $$

where *D* (m) is the aquifer depth, *n* (–) is the porosity, *N* (m) is the annual net recharge, *x*_1_ and *x*_2_ (m) are the distances to the groundwater divide, and *X* (m) is the aquifer width. The transit time was estimated on a 10 × 10 m^2^ grid for three defined hydrological situations based on groundwater depth time series as simulated by WALRUS. If the groundwater depth was greater than 2.25 m (dry conditions), the groundwater was assumed to discharge in the river network only and the aquifer width *X* and distances to the groundwater divide *x*_1_ and *x*_2_ are relatively large. If the groundwater depth was less than 1.25 m, the groundwater was assumed to discharge across the entire drainage network including ditches and trenches, which considerably decrease the values of *X*, *x*_1_, and *x*_2_, and, accordingly, the groundwater transit time *T*.

The metal transit times in the aquifer were estimated by multiplying the groundwater transit times by retardation factors that were derived by Van der Grift and Griffioen (2008). These retardation factors vary spatially depending on the geological formation, infiltration/seepage rates, groundwater depth, and land use. The metal transit times were subsequently scaled up to a 250 × 250 m^2^ grid by fitting cumulative lognormal distributions on the 10 × 10 m^2^ resolution transit times (cf. Wörman et al. 2002). The annual baseflow concentrations were estimated by a flux-weighted convolution of the time series of historic metal concentrations in the leachate with the cumulative lognormal transit time distribution functions.

The metal loads in the quickflow and baseflow were calculated as the product of their respective concentrations and water fluxes as simulated by the WALRUS model. The sum of the metal loads from quickflow and baseflow were transferred to the nearest river network grid cell. The calculated loads and discharge fluxes as simulated by WALRUS were accumulated over the river network and divided by each other to obtain the mean flux-weighted metal concentrations in the groundwater discharged to surface waters in the Dutch part of the Dommel catchment. These flux-weighted concentrations may differ from the actual metal concentrations in the river network since the model calculations do not take the in-channel attenuation of metals, e.g., due to adsorption to bed sediments, into account. In-channel metal attenuation involves many complex hydrological and biogeochemical processes, which are controlled by highly dynamic factors, such as pH, suspended sediment concentration, and bed sediment composition and redox conditions. Metal attenuation was disregarded from the model primarily because of the lack of data about these factors, which is needed to appropriately simulate these processes.

### Model Input

The meteorological forcing of the WALRUS model consisted of daily precipitation and potential evapotranspiration time series. The daily precipitation time series involved time series of precipitation averages for each subcatchment respectively, which were estimated from gridded (1 × 1 km^2^) precipitation data of the Royal Dutch Meteorological Institute (KNMI) for the period 2000–2010 (KNMI 2016). Potential evapotranspiration (PET) time series were constructed as averages for each subcatchment. These averages were estimated by adjusting the daily Makkink (1957) reference evapotranspiration with a combination of crop factors and land use information. The reference evapotranspiration was extracted from the Eindhoven meteorological station for the period 2000–2010. Land use information was derived from the CORINE land cover dataset (Bossard et al. 2000) for Belgium and the STONE database (Van Bakel et al. 2008) for the Netherlands. Table 1 provides an overview of the model input data for the WALRUS and metal transport modules.

**Table 1:** Model input data and parameters

Map or parameter		Location/extent	Reference/source	
1. General catchment characteristics				
Digital Elevation Map (DEM)		Entire catchment	AHN and DHM Flanders (Belgium)^a^	
River network		Entire catchment	TOP10 NL and VHA (Belgium)^b^	
Local-scale drainage (ditches and trenches)		Entire catchment	TOP10 NL^b^	
Soils		Entire catchment	ALTERRA and DOV Flanders (Belgium)^c^	
Land cover		Entire catchment	LGN and CORINE (Belgium)^d^	
Groundwater classes		NL—Dommel	STONE^e^	
2. Meteorology				
Daily (24 h) sum of precipitation (mm)		Entire catchment	KNMI	
Daily (24 h) sum of potential evapotranspiration (mm)		Eindhoven	KNMI	
3. Metal transport				
Aquifer depth		NL—Dommel	REGIS VII.1^f^	
Cd and Zn topsoil concentrations		NL—Dommel	Van der Perk et al. (submitted)	
Soil properties (SOM, clay content, AlFe_ox_, and pH) and topsoil SOC/DOM		NL—Dommel	STONE^e^	
Retardation factors		NL—Dommel	Van der Grift and Griffioen (2008)	
4. Hydrological parameters				
Parameter	Description	Unit	Value/range	Source
*c*_*D*_	Channel depth	mm	800–3100	^g^
*a*_*S*_	SW area fraction	–	0.01–0.05	^h^
*b*	Pore size distribution parameter	–	4.0–5.7	^i^
*Ψ*_*ae*_	Air entry pressure	mm	103–208	^i^
*θ*_*S*_	Saturated soil moisture content	–	0.386–0.424	^i^
*ζ*_1_	Curvature ET reduction function	–	0.02	Brauer et al. (2014a)
*ζ*_2_	Translation ET reduction function	mm	400	Brauer et al. (2014a)
*x*_*S*_	Stage-discharge relation exponent	–	1.5	Brauer et al. (2014a)
*n*	Manning’s roughness coefficient	–	0.03	^j^

^a^5 × 5 m^2^ DEMs were extracted from the AHN (Actual Height Model of the Netherlands) (AHN 2015) and the DHM (Digital Height Model) Flanders (AGIV 2014b) for Belgium. These DEMs were combined, corrected according to the Dutch ordnance, and resampled to 250 × 250 m^2^

^b^TOP10NL: Dutch digital topographic database (Kadaster 2015); VHA: Flemish Hydrographical Atlas (AGIV 2014a)

^c^The soil types for Belgium were extracted from the DOV Flanders (Subsurface Database of Flanders) (DOV 2015) and were reclassified according to the soil types as classified in the Dutch soil maps of ALTERRA

^d^The land cover for Belgian subcatchments were extracted from CORINE (Bossard et al. 2000) and were reclassified according to the land cover as classified in the LGN (Land Use Database of the Netherlands) (Hazeu et al. 2010)

^e^Maps derived from STONE (Van Bakel et al. 2008) were not containing data for built-up areas and areas covered by water. For these areas, data was interpolated from surrounding cells where data was present

^f^The aquifer depth was derived from differences between surface elevations and depths of clay units that were extracted from REGIS II.1 (Vernes et al. 2005)

^g^Channel depths were extracted from the digital elevation model. First, the DEMs of the river, ditch, and trench networks were derived from 5 × 5 m^2^ DEMs. Then the mean of the network elevation over the subcatchments was calculated and resampled to the spatial schematization of the models

^h^Surface water (SW) area fraction was extracted from TOP10 NL by (1) calculating the total SW areas covered by rivers, ditches, and trenches, and (2) calculating the fraction of subcatchment areas covered by SW areas. For the Belgian subcatchments, the SW areas were partly extracted from the VHA and partly taken as the average of SW areas as determined for the Dutch subcatchments

^i^Hydraulic properties for the different soil types were extracted from Clapp and Hornberger (1978) and were averaged for the 44 delineated subcatchments. For peaty soils, hydraulic properties of sandy soils were taken

^j^Based on Manning’s roughness coefficients for clean, straight, natural stream channels (Chow et al. 1988)

#### Calibration and Validation

The WALRUS model was calibrated using time series of daily-observed discharge obtained from the Bossche Broek gauging station (unpublished data, provided by the Dommel Water Authority) (see location G1, Fig. 1). This time series includes measured daily discharges for the period January 2000–February 2015, but contains some data gaps primarily in the periods June–November 2002 and February–October 2003.

Since it was impossible to identify the model separately for each subcatchment (i.e., 44 × 5 = 220 parameters), a parameter regularization method was needed. To this end, the aforementioned calibration parameters—with the exception of *c*_*S*_—were related to the physiographic characteristics of the Dommel catchment resulting in 11 separate parameters to be identified (Table 2). The parameters *c*_*W*_ and *c*_*Q*_ were coupled to groundwater classes derived from the average lowest and highest groundwater levels by distinguishing three classes: wet, intermediate, or dry. The parameters *c*_*V*_ and *c*_*G*_ were coupled to two soil texture classes: sandy and loamy soils. Sandy soils advance quickly to new storage deficit equilibria and are characterized by low flow resistance, short vadose zone relaxation times, and small groundwater reservoir constants. To run the WALRUS model, an area-averaged parameter value was calculated for each parameter and for each subcatchment.

**Table 2:** Initial values and parameter ranges as used in the calibration procedure

Parameter	Units	Initial value	Lower bound	Upper bound
*c*_*W,1*_	mm	110	100	400
*c*_*W,2*_	mm	356	100	400
*c*_*W,3*_	mm	366	100	400
*c*_*V,I*_	h	0.2	0.1	50
*c*_*V,II*_	h	4	0.1	50
*c*_*G,I*_	10^6^ mm h	5	0.1	150
*c*_*G,II*_	10^6^ mm h	10	0.1	150
*c*_*Q,1*_	h	3	1	200
*c*_*Q,2*_	h	12	1	200
*c*_*Q,3*_	h	76	1	200
*c*_*S*_	mm h^-1^	4	0.1	20

*c*_*W*_ wetness index parameter; *c*_*V*_ vadose zone relaxation time; *c*_*G*_ groundwater reservoir constant; *c*_*Q*_ quickflow reservoir constant; *1*, *2*, *3* wet, intermediate, dry; *I*, *II* sand, loam

WALRUS was calibrated using automatic calibration under a cross-validation approach. The initial parameter values and parameter ranges were based on previously reported parameter values (Brauer et al. 2014b) and an a priori model sensitivity analysis. The values and ranges are summarized in Table 2. The cross-validation approach consisted of a calibration and validation for the periods 2000–2006 and 2005–2010, using time series of daily-observed discharge from six gauging stations (unpublished data, provided by the Dommel Water Authority) (see locations G1–G6, Fig. 1) across the Dutch part of the Dommel catchment. The observed discharges at each gauging station were weighted against their upstream areas. The first year of these periods was used as spinning-up period. Several model efficiency criteria were used to test the model’s performance. The best performing parameter set was eventually used for current and future simulations. The automatic calibration was conducted using Parameter ESTimation (PEST) algorithms (Doherty, 2016). In total, two PEST runs were conducted for each calibration period. Between the two PEST runs, water balances were closed to optimize the model performance. Water balances were closed by calculating the mean daily difference between observed and simulated discharge values for the calibration periods 2000–2006 and 2005–2010. The mean daily difference was used as an external flux in WALRUS, representing upward/downward seepage (Brauer et al. 2014a).

To evaluate the outcomes of the metal transport model, simulated flux-weighted concentrations in the discharged runoff were compared with the medians and means of observed Cd and Zn concentrations in the Dommel River. To this end, observations of the period 2001–2010 were used for nine different measurement locations (unpublished data, provided by the Dommel Water Authority) (see locations M1–M9, Fig. 1) that are located in the Dommel River. Since external fluxes from Belgium were not taken into account, boundary conditions were imposed for the Dutch-Belgian border. For the boundary conditions in the main branch of the Dommel river at the Dutch-Belgian border, a bias-corrected (Ferguson, 1986) rating curve between observed Cd and Zn concentrations at the border station (measurement location M10; see Fig. 1) and simulated discharge values was established. The estimated metal concentrations were multiplied by the simulated discharge to obtain the metal loads at the Dutch-Belgian border. For the other border-crossing rivers, no measurements were available. For these rivers, boundary conditions were imposed by using simulated quick- and baseflow metal concentrations from the first Dutch subcatchments, located directly downstream of the Belgian subcatchments. These metal concentrations were used to estimate the total loads that are transferred to the Belgian part of the river network.

### Climate Scenarios

To assess the potential impacts of climate change on metal transport towards surface waters, the hydrology and metal transport were simulated for the periods 2000–2010 (“baseline”) and 2090–2099 (“future”). To simulate future impacts, the WALRUS and Century models were fed with three scenarios: NoCC (i.e., no climate change, based on the current meteorological forcing) and the KNMI’14 climate change scenarios G_H_ and W_H_ (KNMI 2015a). The latter scenarios contain projected changes in climate variables (e.g., precipitation) for two different time horizons, 2050 (2036–2065) and 2085 (2071–2100), and are based on the global climate models as used for the 5th IPCC Assessment report (IPCC 2013). The G_H_ and W_H_ scenarios represent moderate to high global temperature increases and a high likelihood of changes in global air circulation patterns. For the representation of the G_H_ and W_H_ scenarios, future projections of daily precipitation and potential evapotranspiration for 2085 were used (KNMI 2015b; KNMI 2016). The expected changes in precipitation and potential evapotranspiration for the Netherlands are summarized in Table 3.

**Table 3:** Projected future (i.e., 2071–2100) changes in precipitation and potential evapotranspiration for the Netherlands under the climate change scenarios G_H_ and W_H_ (%)

	Precipitation					Potential ET				
	DJF	MAM	JJA	SON	Annual	DJF	MAM	JJA	SON	Annual
G_H_	+12%	+7.5%	−8%	+9%	+5%	+5%	+2%	+8.5%	+3%	+5.5%
W_H_	+30%	+12%	−23%	+12%	+7%	+5%	+3%	+15%	+11%	+10%

## Results

### Current Situation

#### Hydrology

The best performing parameter sets resulting from the WALRUS model calibrations for the two different calibration periods (2000–2006 and 2005–2010, respectively) are given in Table 4. The values of the calibrated parameters do not significantly differ between the two periods. The largest similarities can be found for *c*_*V*_ and *c*_*S*_ in both the values and standard deviations. The low standard deviations for *c*_*V*_ and *c*_*S*_ indicate that the performance of WALRUS is insensitive to changes in the vadose zone relaxation time and the bankfull discharge. The standard deviations of *c*_*Q*_ differ considerably between the two calibration periods, which may indicate that the performance of WALRUS is more sensitive to changes in the quickflow reservoir constant during the period 2000–2006 than during the period 2005–2010.

**Table 4:** Parameter sets and standard deviations resulting from the two different calibrations (Cal. I and Cal. II)

Parameter	Units	Value Cal. I	±*σ* Cal. I	Value Cal. II	±*σ* Cal. II
*c*_*W,1*_	mm	**318**	**135**	219	118
*c*_*W,2*_	mm	**500**	**28**	500	26
*c*_*W,3*_	mm	**161**	**25**	175	25
*c*_*V,I*_	h	**0.2**	**0.04**	0.2	0.06
*c*_*V,II*_	h	**3**	**1**	3	1
*c*_*G,I*_	10^6^ mm h^−1^	**7.0**	**0.9**	6.6	1.1
*c*_*G,II*_	10^6^ mm h^−1^	**6.7**	**4.7**	6.6	6.2
*c*_*Q,1*_	h	**1**	**17**	1	183
*c*_*Q,2*_	h	**18**	**1042**	26	24
*c*_*Q,3*_	h	**123**	**7**	105	19
*c*_*S*_	mm h^−1^	**3**	**0.3**	3	0.3

The parameter set with the best model performance is given in bold

*c*_*W*_ wetness index parameter; *c*_*V*_ vadose zone relaxation time; *c*_*G*_ groundwater reservoir constant; *c*_*Q*_ quickflow reservoir constant; *1*, *2*, *3* wet, intermediate, dry; *I*, *II* sand, loam

**Fig. 2:**
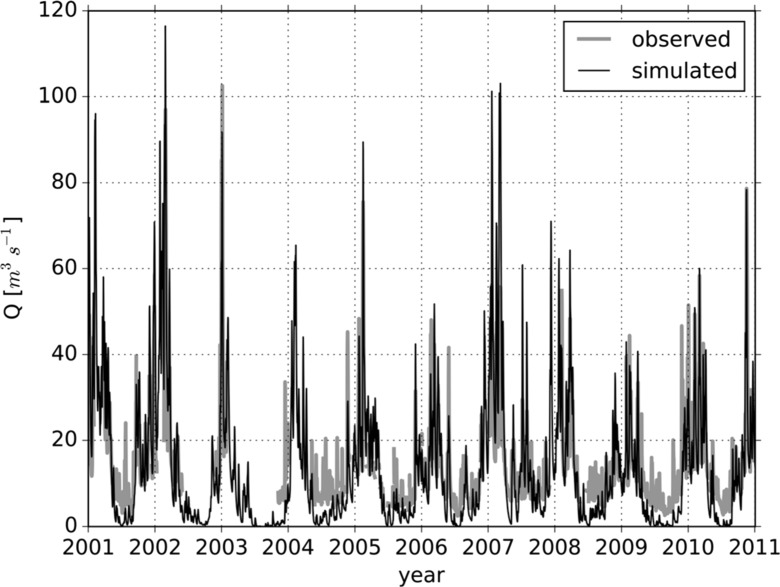
Daily observed versus simulated discharge for the period 2001–2010 at the Bossche Broek gauging station

Table 5 lists the results of the calibration and validation. The model efficiency criteria show consistently better model performances for the period 2000–2006. This can be explained by a combination of missing observation data in the period 2000–2006 during dry periods and an underestimation of simulated discharge during dry periods in the period 2001–2010 (see Fig. 2). Since missing data have not been included in the estimation of model efficiencies, this eventually results in better model performances for the period 2000–2006. A possible explanation for the underestimation of simulated discharge during dry periods is that the discharge in the Dommel River is artificially supplied with water from the Meuse River via the Zuid-Willemsvaart, Wilhelmina, Beatrix, and Eindhoven canals (see Fig. 1) during dry periods (Vroege and Hoijtink 2013).

**Table 5:** Calibration (Cal. P.) and validation periods (Val. P.), and respective NSE (Nash–Sutcliffe Efficiency) (Nash and Sutcliffe 1970), KGE (Kling–Gupta Efficiency) (Gupta et al. 2009), RMSE (root mean square error), and *R* ^2^ (coefficient of determination) values

		NSE (–)	KGE (–)	RMSE (m^3^ s^−1^)	*R* ^2^ (–)
Cal. P. I	2000–2006	0.64	0.62	7.38	0.84
Val. P. I	2005–2010	0.32	0.43	7.85	0.77
Cal. P. II	2005–2010	0.29	0.41	7.99	0.77
Val. P. II	2000–2006	0.63	0.60	7.51	0.84

The best performing calibrated parameter set for the period 2000–2006 was used to simulate current and future discharge. Figure 2 shows the daily simulated and observed discharge time series at the Bossche Broek gauging station for the period 2001–2010, as simulated with this parameter set. As mentioned before, the simulated discharge is underestimated during dry periods. Further, simulated discharge is overestimated during peak flows in some periods, whereas in other periods simulated discharge is slightly underestimated or similar with observed peak flows.

Based on the hydrological simulations, the Dommel catchment can be subdivided into two regions: quickflow-dominated (i.e., where the fraction of quickflow in the total flux to surface waters is higher than 50%) subcatchments (33 subcatchments) and baseflow-dominated subcatchments (11 subcatchments). Table 6 shows the area-averaged annual sums of hydrological fluxes for quickflow- and baseflow-dominated areas under baseline climate conditions. The quickflow-dominated areas are mainly located in the downstream, lower part of the Dommel catchment, whereas the baseflow-dominated areas are generally located in the upstream, higher part of the Dommel catchment. This upstream-downstream division can be explained by the distribution of wet and dry areas in the catchment. In the downstream part of the catchment, relatively more wet floodplains and marshes are present. These wet areas are characterized by high wetness parameters and low quickflow reservoir constants (see Table 4), which causes that most precipitation is directed to quickflow reservoirs. In contrast, the southern, upstream part of the catchment is drier and the contribution of precipitation to the quickflow reservoir is lower. This means that precipitation is mainly directed to the soil-groundwater reservoir, resulting in a larger contribution of baseflow to surface waters.

**Table 6:** Area-averaged annual hydrological fluxes for quickflow- and baseflow-dominated areas under baseline climate conditions and the area-averaged relative flux changes under future climate conditions

		Quickflow-dominated			Baseflow-dominated		
Fluxes	Units	Baseline	G_H_	W_H_	Baseline	G_H_	W_H_
P	mm	842	+2%	+3%	872	+2%	+2%
ETact	mm	509	+4%	+3%	490	+4%	+3%
Q	mm	208	+3%	+6%	260	+2%	+5%
fQS	mm	173	+3%	+6%	59	+9%	+25%
fGS	mm	27	+3%	+4%	193	-1%	-1%

*P* precipitation, *ETact* actual evapotranspiration, *Q* discharge, *fQS* quickflow, *fGS* baseflow

#### Cd and Zn Transport

Table 7 lists the median and mean values of the simulated flux-weighted and observed Cd and Zn concentrations at nine measurement locations in the Dommel River (see Fig. 1). The simulated flux-weighted Cd and Zn concentrations are generally higher than the observed Cd and Zn concentrations. Both the observed and simulated concentrations show a clear negative gradient between upstream and downstream stations, which can be explained by dilution of Cd and Zn during transport through the river network. The simulated flux-weighted Cd and Zn concentrations are higher than the observed Cd and Zn concentrations. For both metals, the overestimation by the model increases more or less consistently from a factor of about 1.2 at the upstream measurement location (M9) to about 3.0 for the most downstream measurement location (M1). This overestimation is most likely due to the fact that in-channel attenuation of metals was not taken into account in the model calculation.

**Table 7:** Median and mean observed and simulated flux-weighted concentrations in the Dommel River

		Cd (mg m^−3^)				Zn (mg m^−3^)			
		Mean		Median		Mean		Median	
Location	Measurement period	Obs	Sim	Obs	Sim	Obs	Sim	Obs	Sim
M1	2001–2007^a^	0.5	1.3	0.4	1.2	47.9	140.7	43.5	134.6
M2	2002–2010^a^	0.4	1.3	0.4	1.2	42.6	142.7	37.0	134.3
M3	2002–2010^a^	0.6	1.6	0.5	1.5	52.7	149.9	44.0	140.4
M4	2004–2010^b^	0.9	1.7	0.8	1.5	66.2	152.6	66.0	145.1
M5	2006–2010^b^	0.9	1.6	0.7	1.6	68.3	155.5	66.0	148.9
M6	2003–2010^b^	0.7	1.7	0.5	1.5	67.8	145.8	59.0	145.3
M7	2003–2010^b^	0.7	1.8	0.7	1.7	74.6	154.4	68.0	151.2
M8	2002–2010^b^	1.5	2.1	1.3	2.1	89.5	165.0	87.0	165.2
M9	2007–2009^b^	3.6	4.5	3.0	4.6	216.9	254.4	220.0	265.7

^a^Bimonthly measurements

^b^Monthly measurements

**Fig. 3:**
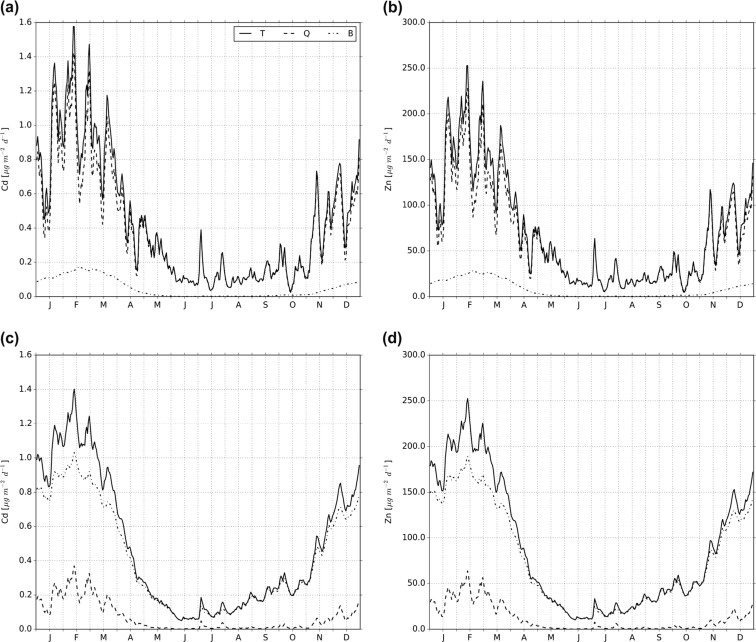
Mean contribution of Cd and Zn loads in quick- and baseflow to the total area-specific Cd and Zn loads in the quickflow dominated area (**a**, **b**) and the baseflow-dominated area (**c**, **d**). *T* total area-specific loads, *Q* contribution of loads in quickflow, *B* contribution of loads in baseflow

Figure 3 shows the mean contribution of Cd and Zn loads in quick- and baseflow to the simulated total area-specific Cd and Zn loads for the downstream and upstream parts of the Dommel catchment for the period 2001–2010. In the downstream part of the catchment, the contribution of the area-specific Cd and Zn loads is dominated by quickflow and baseflow has only a small contribution to the total loads. The highest area-specific Cd and Zn loads occur in the winter period (i.e., up to about 1.6 and 250 μg m^−2^ day^−1^, respectively) and the lowest (i.e., up to about 0.4 and 70 μg m^−2^ day^−1^, respectively) during the summer period. In the upstream part of the catchment, the contributions of Cd and Zn loads are dominated by baseflow, whereas the contribution from quickflow to total loads is small. In this part of the catchment, the highest Cd and Zn loads also occur in the winter period with area-specific loads up to about 1.4 and 250 μg m^−2^ day^−1^, respectively. In the summer period, the area-specific loads are lowest with values up to about 0.2 and 30 μg m^−2^ day^−1^.

**Fig. 4:**
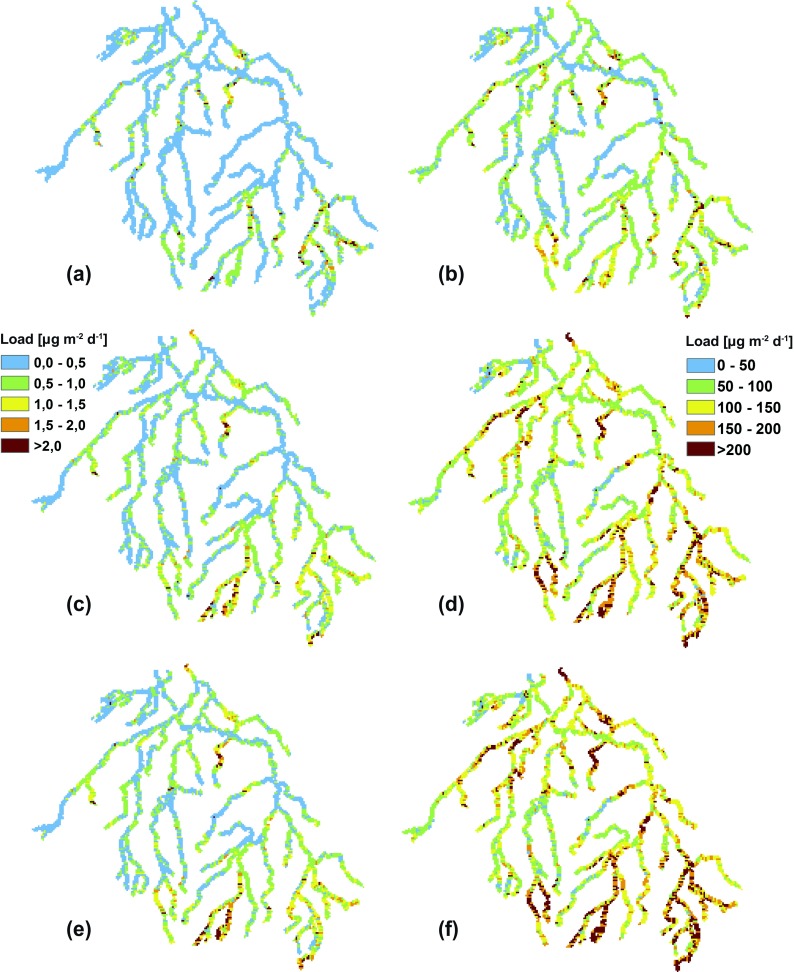
Projected mean area-specific Cd and Zn loads for baseline (**a**, **b**), NoCC (**c**, **d**), and WH (**e**, **f**) climate conditions (μg m^−2^ day^−1^)

Figure 4a and b shows the mean transported area-specific Cd and Zn loads towards the river network in a baseline climate (2001–2010). Cd loads towards the river network are generally highest in the southeastern and north-central part of the Dommel catchment with loads of 2.0 μg m^−2^ day^−1^ or higher (up to 5.7 μg m^−2^ day^−1^). A similar pattern was found for the Zn loads with values of 200 μg m^−2^ day^−1^ or higher (up to 600 μg m^−2^ day^−1^). The high Cd and Zn loads in the southeastern and north-central parts of the Dommel catchment can be mainly attributed to a combination of prevailing quickflow conditions in these areas (i.e., 53% or higher) and high concentrations of Cd and Zn in the fast runoff component, which are primarily derived from inputs from the zinc-ore smelters located in the southeast and the agricultural inputs of Cd and Zn.

### Future Projections

#### Hydrology

**Fig. 5:**
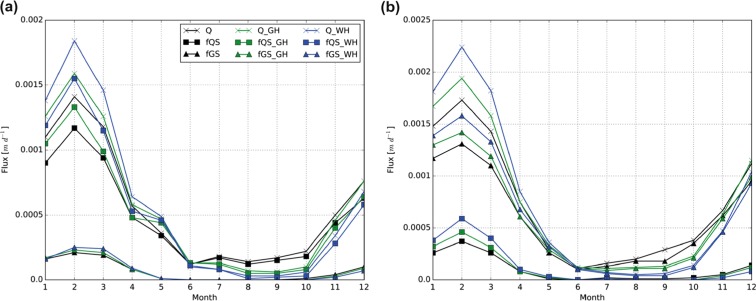
Projected monthly-averaged hydrological fluxes under current and future climate conditions in **a** the quickflow-dominated area and **b** the baseflow-dominated area. *1*–*12* = January–December. *Q* discharge, *fQS* quickflow, *fGS* baseflow, *GH* G_H_, *WH* W_H_

Table 6 summarizes the relative changes in the annual hydrological fluxes under G_H_ and W_H_ climate scenarios for the quickflow- and baseflow-dominated areas in the downstream and upstream parts, respectively, of the Dommel catchment. On an annual basis, both discharge and quickflow are projected to increase under both scenarios and in both areas. The relative changes in baseflow differ between the areas but show similarities between the scenarios. In the downstream area, baseflow is expected to increase by 3% under G_H_ climate conditions and by 4% under W_H_ climate conditions. This indicates that in the quickflow-dominated areas, more groundwater drainage is expected to occur towards the end of the twenty-first century. The projected increase in baseflow is mainly the result of increased precipitation, which is generally greater than the increase in potential evapotranspiration (see Table 3). In the upstream area, the changes in baseflow are expected to be limited with relative decreases of 1% under both climate scenarios. Figure 5 shows the projected changes in discharge, quickflow, and baseflow on a monthly basis for quickflow- and baseflow-dominated areas. During winter, all the hydrological fluxes are projected to increase. Hence, the largest changes in discharge, quickflow (for quickflow-dominated areas), and baseflow (for baseflow-dominated areas) are projected under W_H_ climate conditions. During summer, all fluxes are projected to decrease with the largest decreases under W_H_ climate conditions. The contribution of baseflow in quickflow-dominated areas is, however, expected to remain limited. The projected changes during winter and summer are mainly related to precipitation increases during the winter period and precipitation decreases during the summer period (see Table 3).

#### Cd and Zn Transport

Figure 4c–f shows the mean transported Cd and Zn loads towards the river network under NoCC- and W_H_-climate conditions. Under NoCC-climate conditions, the largest increases in Cd loads (up to 710%) are simulated in the southernmost part and southeastern part of the model area (i.e., region surrounding a Zn smelter where highest metal concentrations in the topsoil are present) with increases from 0.5–1.0 μg m^−2^ day^−1^ to 1.0–2.0 μg m^−2^ day^−1^ or higher (up to 11.4 μg m^−2^ day^−1^) relative to baseline climate conditions. The largest Zn load increases (up to 500%) are simulated for the southeastern, southernmost, northernmost, and western parts of the area with increases from 50–150 μg m^−2^ day^−1^ to 150–200 μg m^−2^ day^−1^ or higher (up to 1100 μg m^−2^ day^−1^). Since hydrological fluxes do not change under NoCC-climate conditions compared to baseline climate conditions, the enhanced loads can be explained by increasing concentrations in the quick- and baseflow components due to a breakthrough of Cd and Zn from the soil system. Hence, the highest increases in quickflow concentrations are generally projected for the western and southeastern parts of the area with Cd increases from 0.8–1.0 mg m^−3^ to 6–10 mg m^−3^ and Zn increases from 100–600 mg m^−3^ to 750–1750 mg m^−3^. The highest increases in baseflow concentrations are generally projected for the northern, north-central, western, and southeastern parts of the area with Cd increases from 0.6–1.2 mg m^−3^ to 4.5–6.0 mg m^−3^ and Zn increases from 150–250 mg m^−3^ to 300–500 mg m^−3^. The magnitude of concentrations in the different flow components indicates that it is likely that quickflow is the largest contributor of Cd and Zn under NoCC-climate conditions. Under the W_H_-climate scenario, the highest Cd and Zn load increases are projected for the same regions as under NoCC-climate conditions. Nevertheless, in comparison with NoCC-climate conditions, relative changes are slightly larger (i.e., up to 820% for Cd and up to 570% for Zn), and loadings are higher (i.e., up to 12.1 μg m^−2^ day^−1^ for Cd and up to 1210 μg m^−2^ day^−1^). This can be mainly attributed to changes in quick- and baseflow. Quickflow is expected to increase in both quickflow- and baseflow-dominated areas (see Table 6), whereas relative changes in baseflow vary per subcatchment. For instance, in the southwestern part of the Dommel catchment, baseflow is expected to decrease with 6% under W_H_-climate conditions, whereas baseflow is expected to increase with 6 and 1% in the southern and southeastern parts of the Dommel catchment, respectively (i.e., where highest Cd and Zn loads increases are projected). Whether quick- or baseflow solely or a combination of these contributors are responsible for load enhancements differs per region. For instance, in the western part of the model area, where the highest increases in quick- and baseflow concentrations are projected under NoCC-climate conditions, both quick- and baseflow increase. This indicates that increases in quick- and baseflow are responsible for the enhancements. In the southern part of the model area, likewise increases in quick- and baseflow are projected. However, the quickflow appears in such small amounts that baseflow can be seen as the only contributor of Cd and Zn. Therefore, it can be concluded that in this part of the area, an acceleration of leaching from groundwater is the main mechanism responsible for the load enhancements.

**Fig. 6:**
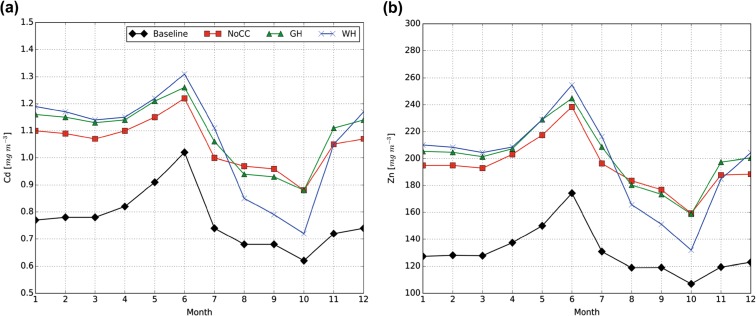
Projected monthly-averaged flux-weighted Cd (**a**) and Zn (**b**) concentrations in the discharged runoff at the outlet of the catchment. *1*–*12* = January–December

Figure 6 shows the projected changes in monthly averaged flux-weighted Cd and Zn concentrations in the discharged runoff as simulated for the outlet of the catchment (i.e., measurement location, M1). The flux-weighted Cd and Zn concentrations are projected to increase in the future under all climate scenarios. The projected monthly average Cd and Zn concentrations in the period 2090–2099 are significantly higher than the current baseline concentrations (two-sample paired *t* test, *α* = 0.05, *p* < 0.001). A two-sample heteroscedastic *t* test showed that the difference is significant for most of the months (*α* = 0.05; *p* generally below 0.001), except for the concentrations for September and October under W_H_-climate conditions. The average difference between monthly average Cd and Zn concentrations under G_H_-climate conditions and NoCC-climate conditions is significantly different from zero (two-sample paired *t* test, *α* = 0.05, *p* < 0.01), whereas the average difference between monthly average Cd and Zn concentrations under W_H_-climate conditions and NoCC-climate conditions does not significantly differ from zero. The monthly average concentrations under a G_H_ climate do not significantly differ from those under the NoCC scenario, except for the Cd concentrations in February (two-sample paired *t* test, *α* = 0.05, *p* = 0.04). The monthly average concentrations under a W_H_ climate are only significantly different from those under a NoCC climate during the months January, February, March, and June (and December only for Zn) (two-sample heteroscedastic *t* test, *α* = 0.05). Compared to the NoCC climate, the lower monthly average metal concentrations in the period August–October under a W_H_ climate are likely to be the result of large variability in precipitation and the associated contribution from quickflow during these months.

On an annual basis, the average flux-weighted Cd and Zn concentrations are likewise significantly higher than current baseline concentrations (two-sample heteroscedastic *t* test, *α* = 0.05, *p* < 0.001) with expected relative increases of approximately 40 and 50%, respectively. Relative to NoCC-climate conditions, Cd and Zn concentrations do not differ significantly from the concentrations under G_H_- and W_H_-climate conditions (two-sample heteroscedastic *t* test, *α* = 0.05).

## Discussion

The simulated current and projected future metal loads and concentrations of our study are generally in line with previous studies in the same region, although details may differ. Van der Grift and Griffioen (2008) simulated Cd concentrations in the Beekloop-Keersop catchment, a small subcatchment in the southern part of the Dommel catchment. They simulated concentrations between 3 μg L^−1^ in 2000 and 5 μg L^−1^ in 2010, which are similar to the flux-weighted Cd concentrations of 4.6 μg L^−1^ (period 2007–2009) simulated in this study for measurement location M9 (i.e., close to the Beekloop-Keersop catchment; see Table 7 and Fig. 1). Our simulated concentrations are also in the range of those reported in the study of Bonten et al. (2012). Bonten et al. (2012) simulated daily Cd concentrations of less than 2 μg L^−1^ (for the period 1993–2001) and daily Zn concentrations of less than 200 μg L^−1^ (for the period 1989–2001) at the outlet of the Dommel catchment during most of the days. We simulated a median Cd concentration of 1.2 μg L^−1^ (period 2001–2007) and a median flux-weighted Zn concentration of 134.6 μg L^−1^ (period 2001–2007) for the same location (measurement location M1; see Table 7 and Fig. 1).

Our results show that future climate change will increase the Cd and Zn loadings towards surface waters. This finding is in line with the study by Joris et al. (2014), who projected increases of 10% in cumulative Cd leaching from the unsaturated zone in the southern part of the Dommel catchment. Nevertheless, future changes differ from those projected in other studies. Visser et al. (2012) projected decreases in Cd and Zn concentrations in the Keersop catchment. These changes were accompanied with decreases in Cd concentrations during winter period from 0.6–0.8 μg L^−1^ under current conditions to 0.2–0.4 μg L^−1^ under climate change and decreases in Zn concentrations from 30–50 to 20–40 μg L^−1^ under the same conditions. In the summer period, the changes are relatively small due to small concentrations already present in surface waters under current and future climate conditions. The different trends among the different studies can mainly be explained by the different climate input. For instance, Visser et al. (2012) projects decreases in discharge and groundwater drainage, meaning less leaching will appear. Joris et al. (2014) projects increases in precipitation and potential evapotranspiration, which results in higher groundwater levels and thus in higher leaching rates.

Future increases in loads and concentrations as a result of changing climate conditions are large compared to current conditions, but limited to the expected changes under NoCC-climate conditions. This indicates that climate change is only a small accelerating factor in the leaching of heavy metals from soil systems. It is likely that concentrations and loads will increase further in the twenty-second century. This can be explained by the combination of projected decreases in Cd and Zn leaching from the topsoil (Van der Perk et al. submitted), and increasing Cd and Zn concentrations in baseflow due to breakthrough of heavy metals. This means that the bulk of heavy metals will still be present in the soil system at the end of the twenty-first century. The combination of expected impacts of climate change on leaching and transport of heavy metals and increasing leaching rates as a result of breakthrough will eventually affect human health and ecosystems. According to European water quality standards, the annual average Cd and Zn concentrations are not allowed to exceed 0.08 and 200 μg L^−1^, respectively (Overheid 2016). This means that the current period Cd concentrations at the outlet of the Dommel catchment (i.e., at measurement location M1) are already far above water quality standards with observed and simulated median flux-weighted Cd concentrations of 0.4 and 1.2 μg L^−1^, respectively. Our results suggest that future Zn concentrations in surface waters will exceed the quality standards as well.

## Conclusions

A semi-distributed hydrological model and a metal transport model were applied to the Dommel catchment to investigate the impact of climate change on future hydrological fluxes and related metal transport pathways in lowland catchments. The hydrological and soil organic matter models, which were implemented in the metal transport model, were forced with future climate change projections.

The outcomes indicate that Cd and Zn loads to surface waters and mean flux-weighted concentrations in the discharge runoff will increase towards the end of the twenty-first century as a result of breakthrough of Cd and Zn in the soil system. Hence, relative changes in daily Cd and Zn loads are expected with relative increases up to 820 and 570%, respectively. Relative changes in Cd and Zn flux-weighted concentrations are in the order of 40 and 50%, respectively. Future climate change is expected to result in increased discharge in winter and decreasing discharge during summer. These changes result eventually in the acceleration of leaching, leading to higher Cd and Zn loads. As a result of climate change, flux-weighted Cd and Zn concentrations increase during early summer and decrease during late summer and early autumn under the most extreme climate scenario. The latter is likely the result of large variability in precipitation and the associated contribution of the quick runoff components to the total runoff during these months.
